# A Missed Opportunity: Understanding the Value of Documenting Occupational Exposure to Carcinogens in Adults With Acute Leukemia

**DOI:** 10.6004/jadpro.2016.7.5.5

**Published:** 2016-07-01

**Authors:** AnnMarie Lee Walton,1, Ashley Leak Bryant,1, Bob Wong,2, Kathi Mooney,2

**Affiliations:** 1The University of North Carolina at Chapel Hill School of Nursing, Chapel Hill, North Carolina; 2The University of Utah College of Nursing, Salt Lake City, Utah

Individuals with occupational exposures to carcinogens are at increased risk for leukemia (Mills, Dodge, & Yang, 2009). Blair et al. (2001) found an increased risk of leukemia for those working in particular industries or occupations and used prior studies to illuminate suspected carcinogens of concern: agricultural service industries (pesticides [Brown et al., 1990]); nursing, health-care workers (ionizing radiation, antineoplastic drugs [Skov et al., 1992], formaldehyde, and unidentified infectious agents); janitors; cleaners (cleaning chemicals and pest control products); those in plumbing, heating, and air-conditioning (asbestos [Schwartz et al., 1988]); and sale of nondurable goods (like paint and varnishes).

With regard to acute myelogenous leukemia (AML) in particular, Tsai et al. (2014) found that construction, crop production or support activities for agriculture and forestry, and animal slaughtering and processing were the occupations most likely to pose a risk for AML (matched odds ratios ranged from 1.13–2.09). Agricultural workers, fishers and fishing workers; nursing, psychiatric, and home health aides; as well as janitors and building cleaners were the occupations at highest risk for AML (matched odds ratios ranged from 1.54–2.02; Tsai et al., 2014).

Benzene and asbestos were the suspected leukemogens in construction (Luckhaupt et al., 2012), pesticides and solvent exposures were of concern for those in agriculture and forestry, and exposure to retroviruses is the concern for those in animal slaughtering and processing, although the authors warned that the evidence of how animal viruses impact human cells needs further study (Tsai et al., 2014). For fishermen, the concern was contaminants (like pesticides) found in fish, as well as stressful sleeping and working conditions (Roberts, Rodgers, & Williams, 2007).

For those in nursing and home health, the concern was for viral exposure and exposure to infectious agents in bodily fluids as well as antineoplastic agents (Skov et al., 1992).

Finally, for those in cleaning occupations, the concern is for exposure to formaldehyde, acetone, sodium hypochlorite, borates, and morpholine (although only formaldehyde is a known leukemogen) as well as pesticides applied in buildings (Blair et al., 2001; Charles, Loomis, & Demissie, 2009).

These studies provide descriptive and correlational data to investigate further the impact of variations in occupational exposure to carcinogens. Yet occupational exposure histories are infrequently conducted as part of an oncology patient’s history and physical, making it difficult for researchers to understand as much as we can about occupational exposures to carcinogens and leukemia. This is a missed opportunity by health-care professionals in recognizing and valuing the importance of documenting an occupational exposure.

We investigated acute leukemia cases (AML and acute lymphocytic leukemia [ALL]) treated at a large comprehensive cancer center in North Carolina from 2007 to 2010. We set out to explore how many of the patients with acute leukemia were in high-risk occupations with documented occupational exposures.

## METHODS

This was a retrospective study that included a convenience sample of individuals diagnosed with acute leukemia at a large regional cancer center in North Carolina. Expedited institutional review board approval was obtained because the data were pulled from existing electronic health records (EHRs) and entered into a database.

**Participants**

Patients aged 18 or older with a diagnosis of acute leukemia receiving care between 2007 and 2010 were identified from the Carolina Data Warehouse for Health (CDW-H). The CDW-H was initiated on July 1, 2004, and is a central repository including clinical, research, and administrative data for patients receiving services at a large cancer center in North Carolina.

Initially, 508 potential patient records were identified. Of them, 184 were diagnosed outside of our study dates (2007–2010) or before 18 years of age. A total of 97 had a diagnosis other than AML or ALL, 72 received their treatment outside of the study center, and 40 were excluded for either insufficient clinical documentation or leukemia secondary to another malignancy. This study included 115 patients, older than 18 years of age at the time of diagnosis with a confirmed diagnosis of AML or ALL who received treatment at a large cancer center in North Carolina between 2007 and 2010.

**Data Collection**

The first two authors (ALW and ALB) developed and entered all data into a database, which captured information about gender, age at diagnosis, current age, race/ethnicity, marital status, insurance status, diagnosis date, type of leukemia, subtype of leukemia, occupation, whether pesticide exposure was assessed or other documentation of occupational exposure was made, number of visits to the emergency department (ED), number of visits to the hospital, number of visits to the clinic, whether or not hematopoietic stem cell transplant (HSCT) was mentioned as a treatment option, whether or not the patient was still in treatment, whether or not the patient had ever achieved remission, whether or not the patient had relapsed, whether or not the patient was deceased at the time of data collection, reason for death, and finally days from diagnosis to death.

Occupations were captured in free text in the database and then coded into 1 of 13 codes, including 1 for not obtained and 1 for unknown. Furthermore, each patient’s occupation was coded as to whether or not there was an increased risk for leukemia based on industry or occupation using the findings of Blair et al. by Standard Industrial Classification Code or Dictionary of Occupational Title (Blair et al., 2001). We also classified military personnel as an employment associated with leukemia based on classifications found in the peer-reviewed Cancer Research Program Fiscal Year 2012 Report to Congress (U.S. Army Medical Research and Material Command, 2012), as military personnel were not a focus of the Blair et al. study.

**Analysis**

As this was a descriptive study, power analysis was not undertaken prior to the start of the study. The Statistical Package for the Social Sciences (SPSS) version 22 was used to code all responses, conduct all data analyses, and compute all summary scores where appropriate (IBM Corporation, 2013). Descriptive statistics including frequency counts and percent statistics were computed for the demographic variables.

## RESULTS

**Sample**

Initially, 508 potential patient records were identified. Of them, 184 were diagnosed outside of our study dates (2007–2010) or before 18 years of age. A total of 97 had a diagnosis other than AML or ALL, 72 received their treatment outside of the study center, and 40 were excluded for either insufficient clinical documentation or leukemia secondary to another malignancy. Of the 115 patients remaining, 57 were women, and 58 were men (age range, 18–82 years). There were 50% (n = 57) non-Latino whites, 20% (n = 23) blacks or members of another race, and 30% (n = 34) Latinos; one patient of an unknown race was included. The majority of patients were married/partnered (73%), and 75% had either no insurance or public insurance (Table 1).

**Table 1:**
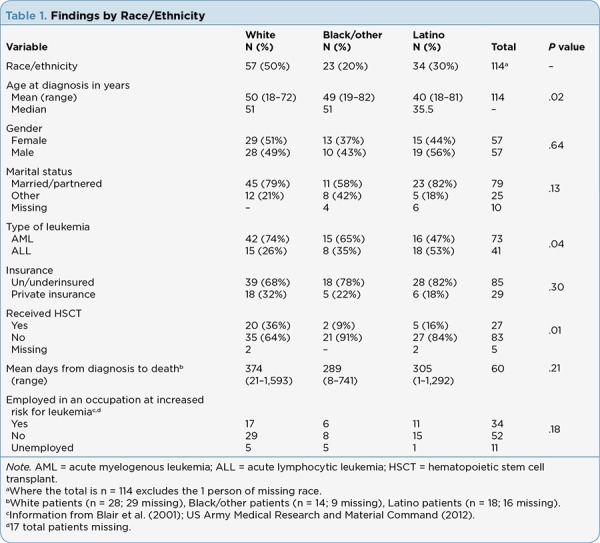
Findings by Race/Ethnicity

**Occupation**

Occupation was noted for 98 of the 115 patients in this sample (Table 2). Of the 17 patients missing occupational data, 10 were women. Although occupation was commonly reported, an assessment of occupational exposures to carcinogens was found in the medical record of only two patients (pesticides for a farm worker and asbestos for a factory worker). Our analyses showed that 35% of our sample for whom occupation was known were at increased risk for leukemia according to their industry or occupational code (Blair et al., 2001).

**Table 2:**
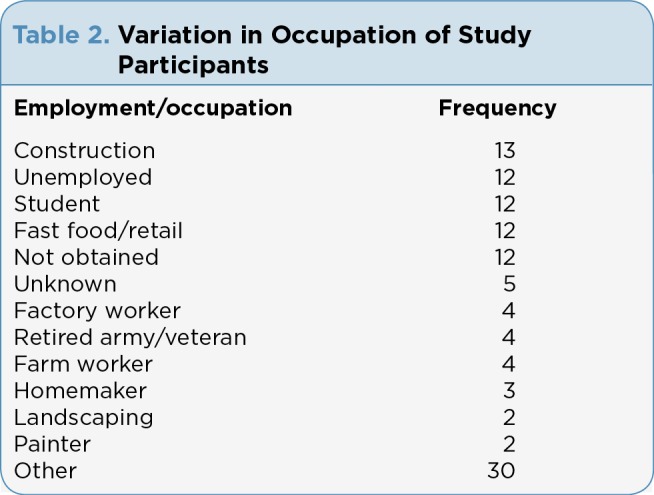
Variation in Occupation of Study Participants

## DISCUSSION

Although it was difficult to answer our original research question with the lack of occupational health information collected, several interesting findings emerged with regard to race/ethnicity (Table 1). Significant differences in age at diagnosis and type of leukemia were found by race/ethnicity. Latinos were younger at diagnosis, with a mean age of 40 (range 18–81) vs. 49 for blacks/members of other races (range, 19–82) and 50 for whites (range, 18–72; *p* = .02). Latinos were more likely to have ALL than were members of other races, with 18 (53% of ALL cases) for Latinos vs. 8 (35% of ALL cases) for blacks/members of other races and 15 (26% of ALL cases) for whites (*p* = .04). Latinos were less likely than non-Latino whites (although slightly more likely than blacks and those of other race/ethnicities) to undergo HSCT. Whites were more likely to receive HSCT (20 [36%]), than Latinos (5 [16%]) and blacks/members of other races (2 [9%], *p* = .01). Nonsignificant statistical differences existed in gender, insurance status, number of patients deceased at the time of the study, length of time from diagnosis to death, and whether or not HSCT was discussed as a treatment option.

More of this sample were Latino than may have been expected compared with the National Cancer Institute’s Surveillance, Epidemiology, and End Results Program (SEER) data on leukemia incidence. According to SEER from 2008 to 2012, the incidence rates by ethnicity and gender per 100,000 were 17.9 male, 10.9 female for whites, 13.5 male and 8.5 female for blacks, and 12.6 male and 8.9 female for Hispanics (National Cancer Institute, 2013). One plausible explanation for this difference is that the state of North Carolina, where this research was conducted, has one of the fastest-growing Hispanic populations in the United States, up 120% from 2000 to 2011 (Brown & Lopez, 2013).

It is worthy to note that more of every racial/ethnic group were un/underinsured than would have been expected compared with insurance data for the state of North Carolina. One plausible explanation may be the institution where this research took place is a not-for-profit health-care system owned by the state. As such, we may see more un/underinsured people than other institutions in our state.

In fiscal year 2010, the hospital system within which the cancer center is located provided $283 million in uncompensated care, which includes indigent care, bad debts, and care costs not reimbursed by Medicare or Medicaid. Uncompensated care was expected to exceed $300 million in the hospital system in fiscal year 2011 (University Gazette, 2011). This particular hospital system was also recognized for providing charity-care levels that exceeded the cost of living for its region (Linker, 2010). In this study, 39 whites (68%), 18 (78%) blacks/members of other races, and 28 (82%) Latinos were un/underinsured. North Carolina state data from 2010 to 2011 illustrated the percentages of each of those same racial/ethnic groups that were uninsured and found that 14.5% of all whites in the state, 41% of all blacks/members of other races, and 41% of Latinos were uninsured (North Carolina Institute of Medicine, 2013).

Extracting data on occupation itself and then determining whether a patient was in an occupation at increased risk for acute leukemia was challenging. The codes that we initially chose for occupations did not match those used by Blair et al., and we compared ours against those they deemed to be at higher risk for any leukemia. There was also variation of risk within our codes, which made it necessary to recode to determine whether there was risk per the Blair article.

For example, housekeeping was complicated. We listed housekeeping under "Other" in Table 2. In our sample, one person cleaned hospitals (considered increased risk per the Blair article), one person cleaned homes (considered not increased risk per the Blair article), and yet another had just "housekeeping" listed as occupation by the provider. Housekeeping in private homes vs. in industry/lodging is associated with a different risk per the Blair article and makes meaningful results challenging.

Determining the set of codes to use was also challenging. We used those Blair provided as being at higher risk for all leukemia, although some of the subanalyses used in the Blair article broke out histologic type, and some work published after our study was complete conducted analyses for particular subtypes as well (Tsai et al., 2014).

Finding only two occupational exposure assessments completed in the workups of 115 patients with leukemia demonstrates the lack of awareness by clinicians of the potential value in collecting this information. Even though 30% (n = 34) of the total sample were in occupations at increased risk for leukemia, in only two charts were there documented exposures.

The questions posed to obtain this information from the individual patient are unknown and underscore that oncologists and advanced practice oncology nurses may not know what to ask. Wider distribution of a resource published by the Agency for Toxic Substances & Disease Registry (2001) called the "I PREPARE," a pocket guide card for primary care providers, may be beneficial for oncologists as well and provide a practical and clinically relevant tool to assess environmental exposures and contribute to the body of knowledge for research (Paranzino, Butterfield, Nastoff, & Ranger, 2005).

The tool was tested and revised based on the input of 159 health-care providers in 2004 and was developed in response to the findings that little time was spent on occupational health in the nursing or medical curricula despite the Institute of Medicine’s strong urgings to the contrary (Paranzino et al., 2005). The tool cues the provider to "Investigate potential exposures"; ask questions about "Present work," "Residence," "Environmental concerns," "Past work," and "Activities"; as well as provide "Referrals and Resources" and "Educate" the patient on strategies to prevent or minimize exposures. Examples of questions in each of those areas can be found in Table 3, and the full PDF is available on the Agency for Toxic Substances and Disease Registry’s website (http://www.atsdr.cdc.gov/asbestos/site-kit/docs/IPrepareCard.pdf).

**Table 3:**
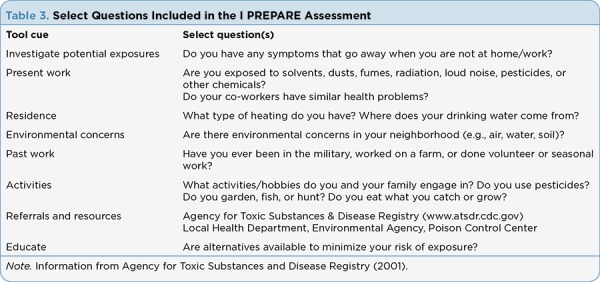
Select Questions Included in the I PREPARE Assessment

Although this tool has not been trialed in the oncology setting, it seems that each of the areas would contribute not only to the data that can be accessed by researchers but to the quality of care patients receive and education for working more safely. In this era, which emphasizes the importance of the learning health system, utilizing the EHR to inform and improve outcomes in patients with cancer, it is paramount that oncology providers understand their significant role in the careful documentation of environmental exposures and the impact that documentation has on data for research. As EHRs are further customized, tools that allow clinicians to quickly collect relative exposure data should be incorporated and will be invaluable to the study of occupational exposures to carcinogens.

**Acknowledgements**

Dr. Walton was supported by the National Institute of Nursing Research of the National Institutes of Health under Award Numbers T32NR007091 and T32NR013456. Dr. Walton was also supported by the Doctoral Scholarship in Cancer Nursing Renewal DSCNR-13-276-03 from the American Cancer Society. Dr. Bryant was supported by the National Cancer Institute of the National Institutes of Health under Award Number R25CA116339 (Bryant) and NCI 5K12CA120780-07 (Bryant). This project was also supported by the NC TraCS Institute, NIH Clinical and Translational Science Award from the National Center for Research Resources UL1TR001111.
